# Impfung gegen coronavirus disease 2019 (COVID-19)

**DOI:** 10.1007/s00391-022-02102-x

**Published:** 2022-09-16

**Authors:** Birgit Weinberger

**Affiliations:** Institut für Biomedizinische Alternsforschung, Universität Innsbruck, Rennweg 10, 6020 Innsbruck, Österreich

**Keywords:** SARS-CoV‑2, SARS-CoV‑2-Varianten, Neutralisierende Antikörper, Immunogenität, Impfempfehlungen, SARS-CoV‑2, SARS-CoV‑2 variants, Antibodies, neutralizing, Immunogenicity, vaccine, Vaccination recommendations

## Abstract

Die klinischen Präsentationen von Infektionen mit SARS-CoV‑2 („severe acute respiratory syndrome coronavirus 2“) sind sehr heterogen, und das Risiko für einen schweren Verlauf steigt mit zunehmendem Alter deutlich an. Ältere Erwachsene sind deshalb eine wichtige Zielgruppe für Impfungen. In Europa sind derzeit 2 mRNA-Impfstoffe, 2 adenovirale Vektorimpfstoffe und ein Proteinimpfstoff für ältere Erwachsene zugelassen. Die Immunogenität und klinische Wirksamkeit dieser Impfstoffe waren in den ersten Zulassungsstudien bei älteren Erwachsenen ähnlich oder nur geringfügig niedriger als in jüngeren Altersgruppen. Allerdings nehmen die Konzentration neutralisierender Antikörper und der Schutz vor Infektion im Laufe der Zeit deutlich ab und sind gegen Virusvarianten, besonders gegen Omicron, stark reduziert. Der Schutz vor schwerer Krankheit und Hospitalisierung ist jedoch langlebiger und nach 3 Impfdosen im Schema 2 + 1 auch für Omicron gegeben. Weitere Auffrischungsimpfungen sind derzeit für Risikopatienten, insbesondere für ältere Erwachsene, empfohlen. Bezüglich der konkreten, aktuell gültigen Empfehlungen für verschiedene Alters- und Risikogruppen wird auf die Bekanntmachungen der nationalen Impfgremien verwiesen.

Alle derzeit verfügbaren Impfstoffe beruhen auf der ursprünglichen Virusvariante. Impfstoffe, die an die neuen Virusvarianten angepasst sind, werden derzeit erprobt, und ein zeitnaher Wechsel zu diesen Impfstoffen ist wahrscheinlich. Allerdings schreitet auch die Veränderung der Viruspopulationen voran, sodass voraussichtlich eine kontinuierliche Weiterentwicklung der Impfstoffe notwendig sein wird.

## Abstract (en)

The clinical presentation of infections with severe acute respiratory syndrome coronavirus 2 (SARS-CoV‑2) is very heterogeneous and the risk of a severe course clearly increases with age. Therefore, older adults are an important target group for vaccinations. Several vaccines are currently licensed in Europe for older adults, namely two mRNA vaccines, two adenoviral vector vaccines and a protein-based vaccine. The immunogenicity and clinical efficacy of these vaccines in the first approval trials were equal or only slightly reduced for older adults compared to younger age groups; however, the concentration of neutralizing antibodies and protection against infection greatly declined over time and the latter is substantially reduced for virus variants, particularly for the Omicron variant. Nevertheless, protection against severe disease and hospitalization is maintained at a high level for longer time periods, and after three vaccine doses (2 + 1 schedule) also for the Omicron variant. Additional booster vaccinations are currently recommended for patients with risk factors, especially older adults. With respect to the currently valid recommendations for different age and risk groups, the publications and notifications of the national vaccine advisory bodies should be referred to.

All currently available vaccines target the original virus strain. New vaccines, which are adapted to virus variants are currently being developed and tested, and it is highly likely that they will be used in the near future; however, viral evolution is ongoing and a continuous development of adapted vaccines will probably be necessary.

Infektionen mit SARS-CoV‑2 („severe acute respiratory syndrome coronavirus 2“) und die damit verbundene Erkrankung COVID-19 („coronavirus disease 2019“) stellen seit dem ersten Auftreten dieser Erkrankung eine große Herausforderung für jeden einzelnen, für das Gesundheitswesen und die gesamte Gesellschaft dar. Da ältere Personen überproportional häufig schwer erkranken, sind sie eine wichtige Zielgruppe für Präventionsstrategien, besonders für Impfungen. Dieser Beitrag behandelt die Impfstoffe gegen SARS-CoV‑2/COVID-19, die derzeit in Europa zur Verfügung stehen.

## Impfstoffe

Die klinischen Präsentationen von SARS-CoV‑2-Infektionen reichen von asymptomatischen bis hin zu letalen Verläufen, wobei das Risiko, im Fall einer Infektion schwer zu erkranken oder zu versterben, mit zunehmendem Alter deutlich ansteigt. Personen im Alter von 75 bis 84 Jahren haben bei vorliegender SARS-CoV‑2-Infektion ein 8fach erhöhtes Risiko für eine Hospitalisierung und ein 140-fach erhöhtes Risiko zu versterben als die Referenzgruppe (18 bis 29 Jahre). Für Personen ≥ 85 Jahren ist das Risiko sogar um das 10-Fache bzw. 330-Fache erhöht [4]. Auch Personen mit chronischen Grund- und Autoimmunerkrankungen, Krebspatienten, Patienten unter Immunsuppression, Menschen mit Diabetes und stark Übergewichtige sind stärker gefährdet [2]. Viele ältere Personen weisen zusätzlich einen oder mehrere dieser Risikofaktoren auf. Nichtpharmazeutische Interventionen können das Infektionsgeschehen bremsen, sind jedoch mit großen persönlichen, gesellschaftlichen und finanziellen Einschränkungen verbunden. Die effektivste Maßnahme, um schwere Erkrankungen zu verhindern, ist die Impfung.

Im Dezember 2020, weniger als ein Jahr nach der Identifizierung von SARS-CoV‑2, erhielten die ersten Impfstoffe gegen dieses Virus eine bedingte Zulassung in der Europäischen Union, und in vielen Ländern starteten breit angelegte Impfkampagnen.

Alle bisher zugelassenen Impfstoffe (Tab. 1) basieren auf dem ursprünglichen Virusstamm und werden intramuskulär (i.m.) verabreicht.

**Table 1:** 

Name	Handelsname	Hersteller	Art des Impfstoffs	Antigen	Dosen (Abstand in Wochen)
BNT162b2	Comirnaty	BioNTech/Pfizer	Modifizierte mRNA in Lipidnanopartikeln	Spike-Protein	2 (3)
mRNA-1273	Spikevax	Moderna	Modifizierte mRNA in Lipidnanopartikeln	Spike-Protein	2 (4)
AZD1222	Vaxzevria	Oxford/AstraZeneca	Schimpansen-Adenovirus-Vektor	Spike-Protein	2 (4–12)
Ad26.COV2‑S	Jcovden	Janssen/Johnson & Johnson	Adenovirus-26-Vektor	Spike-Protein	1
NVX-CoV2373	Nuvaxovid	Novavax	Rekombinantes Protein; Matrix M	Spike-Protein	2 (3)
VLA-2001	–	Valneva	Ganzvirus, inaktiviert; Aluminiumhydroxid, CpG1018	Ganzvirus	2 (4)

*SARS-CoV‑2* „severe acute respiratory syndrome coronavirus 2“

## Klinische Wirksamkeit

### Zulassungsstudien

Die Basis für die Zulassung von BNT162b2, mRNA-1273, AZD1222, Ad26.COV2‑S und NVX-CoV2373 bilden randomisierte, placebo- oder komaparatorkontrollierte Studien mit jeweils 10.000 bis 45.000 Teilnehmern.

Für BNT162b2 ist die klinische Wirksamkeit bei Geimpften im Alter ≥ 55 und ≥ 65 Jahren identisch zur Situation jüngerer Erwachsener. Die Wirksamkeit von mRNA-1273 ist bei Personen ≥ 65 Jahren geringfügig niedriger als bei jüngeren Erwachsenen. Die Wirksamkeit beider Impfstoffe scheint auch bei Teilnehmern ≥ 75 Jahren gegeben zu sein, allerdings waren in beiden Studien nur sehr wenige Teilnehmer über 75 Jahre alt, und deshalb ist die statistische Aussagekraft für diese Gruppe gering. Werden diese Daten mit anderen Impfstoffen verglichen, zeigt sich ein sehr positives Wirksamkeitsprofil für ältere Personen. In den klinischen Zulassungsstudien für AZD1222 waren nur sehr wenige Teilnehmer über 55 Jahre alt, sodass keine statistisch belastbaren Aussagen über die klinische Wirksamkeit bei älteren Erwachsenen getroffen werden konnten. Immunogenitäts- und Sicherheitsdaten waren für Personen ≥ 65 Jahre ähnlich gut wie bei jüngeren Erwachsenen [12, 25]. Die klinische Wirksamkeit von Ad26.COV2‑S ist für die beiden Altersgruppen < bzw. ≥ 60 Jahren vergleichbar, tendenziell in der älteren Gruppe sogar höher. In den beiden Phase-III-Studien mit NVX-CoV2373 waren 12 % [9] bzw. 28 % [16] der Teilnehmer älter als 65 Jahre. Die klinische Wirksamkeit ist für beide Altersgruppen vergleichbar. Die genauen Zahlen und die Aufschlüsselung nach Altersgruppen finden sich in Tab. 2.

**Table 2:** 

**BNT162b2**					
Noori et al. [21]	*Alle Teilnehmer*	*16 bis 55 Jahre*	*>* *55 Jahre*	*≥* *65 Jahre*	*≥* *75 Jahre*
KW^a^	95,0	95,6	93,7	94,7	100
95 %-KI	90,0–97,9	89,4–98,6	80,6–98,8	66,7–99,9	−13,1–100
**mRNA-1273 **					
Baden et al. [1], European Medicines Agency [11]	*Alle Teilnehmer*	*18 bis 64 Jahre*	*≥* *65 Jahre*	*≥* *75 Jahre*	
KW^a^	94,1	95,6	86,4	100	
95 %-KI	89,3–96,8	90,6–97,9	61,4–95,2	NE-100	
**AZD1222**					
European Medicines Agency [12], Voysey et al. [25]	*Alle Teilnehmer*				
KW^a^	59,5				
95 %-KI	45,8–69,7				
**Ad26.COV2‑S**					
Sadoff et al. [22]	*Alle Teilnehmer*	*18 bis 59 Jahre*	*≥* *60 Jahre*		
KW^b^	66,9	63,7	76,3		
95 %-KI	59,03–73,40	53,87–71,58	61,58–86,04		
**NVX-CoV2373**					
Dunkle et al. [9]	*Alle Teilnehmer*	*18 bis 64 Jahre*	*Hochrisiko (einschl. ≥* *65 Jahre)*		
KW^a^	90,4	91,5	91,0		
95 %-KI	82,9–94,6	84,2–95,4	83,6–95,0		
Heath et al. [16]	*Alle Teilnehmer*	*18 bis 64 Jahre*	*≥* *65 Jahre*		
KW^a^	89,7	89,8	88,9		
95 %-KI	80,2–94,6	79,7–95,5	20,2–99,7		

*KW* Klinische Wirksamkeit, *KI* Konfidenzintervall, *SARS-CoV‑2* „severe acute respiratory syndrome coronavirus 2“, *NE* non estimable

^a^Symptomatische, laborbestätigte Infektion

^b^Moderate und schwere symptomatische, laborbestätigte Infektion

Für den Impfstoff VLA-2001 liegen keine Wirksamkeitsdaten vor. Sicherheit und Immunogenität von 2 Dosen im Abstand von 4 Wochen wurden in einer Studie mit ca. 3000 Teilnehmern untersucht, wobei als Komparator AZD1222 verwendet wurde. Das Sicherheitsprofil für VLA-2001 war ähnlich bzw. tendenziell besser als für AZD1222. Die Konzentration neutralisierender Antikörper war 2 Wochen nach der 2. Dosis VLA-2001 1,4-mal höher als nach der Impfung mit AZD1222 [13]. Die bedingte Zulassung für VLA-2001 ist auf die Altersgruppe 18 bis 50 Jahre beschränkt, da nur 1 % der Studienteilnehmer älter als 50 Jahre war.

### Bevölkerung

Erste umfassende Daten zur Wirksamkeit in der Bevölkerung stammen aus Israel; dort waren bis Anfang April 2021 bereits 90 % der Bevölkerung im Alter ≥ 65 Jahren mit 2 Dosen BNT162b2 geimpft. Die Wirksamkeit gegen eine asymptomatische Infektion mit SARS-CoV‑2 in der Altersgruppe ≥ 65 Jahren betrug 86 %. Alle anderen Endpunkte und Altersgruppen wiesen eine höhere Wirksamkeit auf, viele davon über 95 %. Eine detaillierte Analyse der ältesten Bevölkerungsteile zeigt keine Unterschiede für die Altersgruppen ≥ 65, ≥ 75 und ≥ 85 Jahre, wobei die Wirksamkeit der Impfung gegen eine Hospitalisierung für alle Altersgruppen 97 % betrug [14]. In der Folge wurden diese Daten in anderen Ländern bestätigt. Die Zulassungsstudien und diese ersten Daten aus der Anwendung der Impfstoffe umfassen nur Beobachtungszeiträume von wenigen Wochen. Dieser Umstand muss beim Vergleich mit anderen Impfstoffen und Studien, die üblicherweise einen Zeitraum von mehreren Jahren einschließen, berücksichtigt werden.

In der weiteren Folge wurde deutlich, dass die Antikörperkonzentrationen im Laufe der Zeit abnehmen. Eine Studie, die in diesem Kontext speziell ältere Erwachsene untersuchte, zeigt, dass Antikörperkonzentrationen, neutralisierende Antikörper und T‑Zell-Antworten 6 Monate nach der Impfung mit BNT162b2 bei älteren Erwachsenen in Pflegeeinrichtungen deutlich niedriger sind als bei jungen Erwachsenen [24]. Neutralisierende Antikörper dienen nach wie vor häufig als Surrogatmarker für die Schutzwirkung, allerdings muss berücksichtigt werden, dass sie zwar wohl entscheidend am Schutz vor Infektionen beteiligt sind, der Schutz vor schwerer Erkrankung jedoch auch maßgeblich durch Gedächtnis-B-Zellen und T‑Zell-Antworten vermittelt wird.

Analysen der klinischen Wirksamkeit über längere Zeiträume sind komplex. In Beobachtungsstudien müssen neben der zeitlichen Dimension auch andere Faktoren berücksichtigt werden. In vielen Ländern wurden unterschiedliche Altersgruppen aufgrund der Priorisierung zu unterschiedlichen Zeitpunkten und teilweise auch mit unterschiedlichen Impfstoffen geimpft. Die epidemiologische Lage war über die Zeit nicht konstant, und es traten – in zeitlicher Abfolge – verschiedene Virusvarianten auf.

## Rolle von Virusvarianten

In Europa war der Wechsel von der ursprünglichen Viruspopulation zu Alpha (Anfang 2021), Delta (Sommer 2021) und Omicron (Jahreswechsel 2021/2022) relevant, wobei in den letzten Monaten nacheinander verschiedene Omicron-Subvarianten definiert wurden (BA.1, BA.2, BA.4, BA.5).

Es wurde in einer Vielzahl von Studien gezeigt, dass impfinduzierte Antikörper speziell die neueren Virusvarianten weniger effektiv neutralisieren können [17, 21]. Eine rezente Studie belegt, dass Antikörper, die durch Impfung oder Infektion mit einer der früheren Varianten induziert wurden, die Omicron-Variante deutlich schlechter neutralisieren können. Im Gegensatz zu den anderen Varianten ist für die Neutralisierung von Omicron die 3. Impfdosis essenziell [8]. Die Virusvariante, die in dieser Studie untersucht wurde, wurde in der Zwischenzeit als BA.1 klassifiziert. In den wenigen Monaten seit der Veröffentlichung setzen sich in vielen Ländern BA.4/BA.5 durch und verdrängen frühere Omicron-Varianten. Auch die Antikörper, die nach einer Impfung und zusätzlicher Infektion mit Omicron BA.1 gebildet werden, können BA.4 und BA.5 nicht vollständig neutralisieren [3]. Es muss also mit Reinfektionen auch nach erst kürzlicher Infektion mit BA.1 gerechnet werden.

In einer Studie aus den USA, die die Wirksamkeit der mRNA-Impfstoffe bis zu 8 Monate nach der Impfung analysierte, sank diese gegen eine Infektion mit SARS-CoV‑2 (asymptomatisch und symptomatisch) im Beobachtungszeitraum von über 90 % auf 70–80 % ab. Die Wirksamkeit war in der Altersgruppe ≥ 65 Jahre nur geringfügig niedriger. Im Beobachtungszeitraum wurde die ursprüngliche Virusvariante zunächst von Alpha und diese dann von Delta abgelöst. Im Gegensatz dazu beträgt die Wirksamkeit gegen Hospitalisierung nahezu konstant rund 90 % und ist ebenfalls für Personen über 65 Jahren nur geringfügig niedriger [18]. Personen, bei denen die Impfung mit 2 Dosen mRNA-Impfstoff mehr als 6 Monate zurückliegt, sind deutlich besser gegen die Delta-Variante geschützt als gegen Omicron. Hier beträgt der Schutz gegen Hospitalisierung nur 57 %. Die 3. Dosis eines mRNA-Impfstoffs erhöht die Schutzwirkung gegen eine Hospitalisierung auf 94 % (Delta) bzw. 90 % (Omicron). Allerdings war der Beobachtungszeitraum nach der 3. Dosis in dieser Studie relativ kurz [23]. In einer Studie aus Katar wurde die Wirksamkeit der mRNA-Impfstoffe gegen Omicron BA.1 und BA.2 untersucht. Diese Studie bestätigt die hohe Wirksamkeit gegen schwere Erkrankungen und Hospitalisierung, obwohl die Wirksamkeit gegen eine Infektion mit Omicron nach 2 Dosen gering ist und nur durch die 3. Dosis verbessert wird [6]. Metaanalysen und systematische Reviews gibt es bisher hauptsächlich für die Delta-Variante [15], und die aktuellen klinischen Daten beziehen sich auf Omicron BA.1 und BA.2. Mit ähnlichen Studien zu BA.4 und BA.5 kann in Kürze gerechnet werden.

Zusammenfassend kann geschlussfolgert werden, dass die Konzentrationen der neutralisierenden Antikörper im Laufe der Zeit abnehmen und ihre Wirksamkeit gegenüber Virusvarianten reduziert ist. Die klinische Wirksamkeit der Impfung gegen asymptomatische und mild symptomatische Infektion nimmt ebenfalls mit der Zeit ab und ist gegenüber Virusvarianten deutlich verringert. Ähnliches gilt auch für die Schutzwirkung einer durchgemachten Infektion gegenüber der Reinfektion. Die Wirksamkeit der Impfung gegen schwere Erkrankungen, Hospitalisierung und Tod ist jedoch langlebiger und auch gegenüber Virusvarianten gegeben.

## Impfstrategien

Nationale Impfstrategien unterscheiden sich im Detail und werden laufend an die epidemiologische Situation, neue Varianten und aktuelle Studienergebnisse angepasst. Deshalb wird für die jeweils aktuell gültigen Empfehlungen auf die nationalen Gremien und deren Publikationen verwiesen (Tab. 3) und im Folgenden lediglich ein Überblick über die grundlegenden Prinzipien gegeben.

**Table 3:** 

Deutschland	https://www.rki.de/DE/Content/Infekt/Impfen/ImpfungenAZ/COVID-19/Impfempfehlung-Zusfassung.html
Österreich	https://www.sozialministerium.at/Corona/Corona-Schutzimpfung/Corona-Schutzimpfung---Fachinformationen.html
Schweiz	https://www.bag.admin.ch/bag/de/home/krankheiten/ausbrueche-epidemien-pandemien/aktuelle-ausbrueche-epidemien/novel-cov/information-fuer-die-aerzteschaft/covid-19-impfung.html

BNT162b2, mRNA-1273 und AZD1222 wurden zunächst als Impfserie mit je 2 Dosen im Abstand weniger Wochen verabreicht. Der Impfstoff Ad26.COV2 wurde ursprünglich als Einzeldosis zugelassen und eingesetzt. Es zeigte sich jedoch bald, dass der Schutz – speziell gegen die Delta-Variante – nach nur einer Dosis unzureichend ist, und es erfolgte in vielen Ländern die Empfehlung, 4 Wochen nach der ersten Dosis Ad26.COV2 eine weitere Dosis, präferenziell eines mRNA-Impfstoffs, zu verabreichen. Im Spätsommer/Herbst 2021 folgten die ersten Empfehlungen für die 3. Impfdosis, zunächst wieder für ältere Erwachsene und Risikogruppen, im weiteren Verlauf für alle. Bei der Definition des optimalen Zeitpunkts für die 3. Dosis stehen sich epidemiologische (z. B. Beginn einer Infektionswelle) und immunologische Überlegungen (längere Abstände für optimale Booster-Antwort) gegenüber. In vielen Ländern wurde zunächst empfohlen, die 3. Dosis 4 bis 6 Monate nach der 2. Dosis zu verabreichen. Inzwischen wird für die mRNA- und Adenovirus-Vektor-Impfstoffe ein 2 + 1 Impfschema (3 Dosen mit zunächst einigen Wochen und dann 6 Monaten Abstand) als vollständige Grundimmunisierung angesehen. Ende 2021 erfolgte die bedingte Zulassung des Impfstoffs NVX-CoV2373, der ebenfalls zunächst in 2 Dosen im Abstand weniger Wochen verabreicht wird, und damit steht auch ein Proteinimpfstoff zur Verfügung. Eine 3. Dosis wird auch für diesen Impfstoff notwendig sein. Die Empfehlung dazu wird erfolgen, wenn ausreichende Daten zur Verfügung stehen. Speziell für den Schutz vor Virusvarianten ist die 3. Dosis entscheidend, weil die Antikörperkonzentrationen durch diese Auffrischung nicht nur erhöht werden, sondern auch eine breitere Immunantwort induziert wird [7, 20].

Derzeit wird eine 4. Dosis, wiederum 6 Monate nach der 3. Impfung in einigen Ländern für Risikopatienten, speziell auch für ältere Erwachsene, empfohlen. In Anbetracht der aktuell steigenden Fallzahlen (Juli 2022) wird diese Empfehlung in der nahen Zukunft voraussichtlich auf weitere Personengruppen ausgedehnt werden.

Seit dem Auftreten der Omicron-Variante und ihrer Fähigkeit, die bestehende Immunantwort besser zu umgehen, wird intensiv über variantenspezifische, angepasste Impfstoffe diskutiert. BioNTech/Pfizer, Moderna und Novavax arbeiten an angepassten Impfstoffen. Dabei werden sowohl Präparate, die nur die Omicron-Variante enthalten, als auch Kombinationen aus Omicron und ursprünglichem Impfstoff getestet. In einer Studie wurde Personen, die bereits 3 Dosen mRNA-1273 erhalten hatten, als 4. Dosis entweder nochmals mRNA-1273 (nur ursprüngliche Variante) oder mRNA-1273.214 (ursprüngliche Variante und Omicron) verabreicht. Die Antikörperantworten gegen die Omicron-Variante waren nach dem bivalenten Booster 1,75fach höher, während die Immunantworten gegen die ursprüngliche Variante vergleichbar waren [5]. BioNTech/Pfizer und Novavax berichten bisher nur in Pressemitteilungen über vielversprechende Ergebnisse ihrer Omicron- oder bivalenten Booster-Impfstoffe.

Allerdings beruhen all diese Impfstoffkandidaten auf der Omicron-Variante BA.1, die in der Zwischenzeit schon wieder verdrängt wurde. Trotzdem sprachen sich die Weltgesundheitsorganisation [26] und das Beratungskomitee der Food and Drug Administration (FDA, USA) [10] im Juni 2022 dafür aus, in Zukunft die Omicron-Variante in Impfstoffen zu berücksichtigen. Wenige Tage später folgte eine Mitteilung der FDA, die Impfstoffherstellern dazu rät, für modifizierte Impfstoffe den bisherigen Komponenten eine an BA.4/5 angepasste Variante hinzuzufügen und so bivalente Impfstoffe (ursprüngliche Variante + BA.4/5) zu generieren [19]. Es bleibt abzuwarten, wie sich diese Entwicklung fortsetzt, und welche Impfstoffe zum Einsatz kommen werden.

## Fazit für die Praxis

- Die Konzentrationen neutralisierender Antikörper und die Wirksamkeit der Impfung gegen eine Infektion mit dem Severe acute respiratory syndrome coronavirus 2 (SARS-CoV‑2) nehmen im Laufe der Zeit ab und sind gegen Virusvarianten niedriger.
- Die Wirksamkeit der Impfung gegen schwere Verläufe, Hospitalisierung und Tod ist jedoch deutlich langlebiger und auch für Virusvarianten gegeben.
- Eine vollständige Grundimmunisierung gegen die Coronavirus disease 2019 (COVID-19) umfasst 3 Impfdosen im Schema 2 + 1. Weitere Auffrischungsimpfungen sind derzeit v. a. für Risikopatienten, insbesondere für ältere Erwachsene, empfohlen.
- Ein Wechsel zu Impfstoffen, die an neue Virusvarianten angepasst sind, ist wahrscheinlich.
- Die aktuell gültigen Impfempfehlungen werden regelmäßig von den zuständigen Behörden veröffentlicht.
